# CBX7 and miR‐9 are part of an autoregulatory loop controlling p16^INK^ ^4a^

**DOI:** 10.1111/acel.12404

**Published:** 2015-09-29

**Authors:** Ana O'Loghlen, Sharon Brookes, Nadine Martin, Valentina Rapisarda, Gordon Peters, Jesús Gil

**Affiliations:** ^1^ Cell Proliferation GroupMRC Clinical Sciences CentreImperial College LondonHammersmith CampusLondonW12 0NNUK; ^2^ Molecular Oncology LaboratoryCRUK London Research Institute44 Lincoln's Inn FieldsLondonWC2A 3LYUK; ^3^ Epigenetics & Cellular Senescence GroupBlizard InstituteBarts and The London School of Medicine and DentistryQueen Mary University of London4 Newark StreetLondonE1 2ATUK; ^4^ Senescence Escape Mechanisms LaboratoryInserm U1052CNRS UMR5286Centre de Recherche en Cancérologie de LyonLyonFrance

**Keywords:** CBX7, miR‐9, p16^INK^^4a^, Polycomb, senescence

## Abstract

Polycomb repressive complexes (PRC1 and PRC2) are epigenetic regulators that act in coordination to influence multiple cellular processes including pluripotency, differentiation, cancer and senescence. The role of PRCs in senescence can be mostly explained by their ability to repress the *INK4/ARF* locus. CBX7 is one of five mammalian orthologues of Drosophila Polycomb that forms part of PRC1. Despite the relevance of CBX7 for regulating senescence and pluripotency, we have a limited understanding of how the expression of CBX7 is regulated. Here we report that the miR‐9 family of microRNAs (miRNAS) downregulates the expression of CBX7. In turn, CBX7 represses miR‐9‐1 and miR‐9‐2 as part of a regulatory negative feedback loop. The miR‐9/CBX7 feedback loop is a regulatory module contributing to induction of the cyclin‐dependent kinase inhibitor (CDKI) p16^INK^ ^4a^ during senescence. The ability of the miR‐9 family to regulate senescence could have implications for understanding the role of miR‐9 in cancer and aging.

## Introduction

The majority of the mammalian genome is transcribed but only 2% of it is translated. The differential between the transcribed and translated genomes highlights the importance of noncoding RNAs in mediating and regulating physiological processes (Amaral *et al*., 2008). MicroRNAs (miRNAs) are small noncoding RNAs that bind to complementary sequences in their target mRNAs and either block their translation or promote their turnover (Bartel, 2004, 2009). As each miRNA can target multiple mRNAs, they can have profound effects on the patterns of gene expression and individual miRNAs have been shown to influence a broad range of cellular processes, including development, cancer, senescence and aging (Ambros, 2004). This functional pleiotropism has given rise to the concept of ‘miRNA expression signatures’, which differ depending on the tissue, cell type and the pathology. For example, miR‐9 is expressed at high levels in neural tissues and is essential for the development of the brain (Delaloy *et al*., 2010; Uchida, 2010). miR‐9 plays a critical role in regulating the proliferation of neural progenitors and, not surprisingly, has been implicated in the development of different types of brain tumour, such as medulloblastoma and glioblastoma (Ferretti *et al*., 2009; Malzkorn *et al*., 2010). Several miR‐9 target genes have been described that might play a role in this context. One of the most prominent is the nuclear receptor NR2E1 (also called TLX), which is essential for the proliferation and self‐renewal of neural stem cells (NSC) and brain tumour stem cells (BTSC) (Liu *et al*., 2010; Zhu *et al*., 2014). Besides its role in the brain, miR‐9 also influences normal physiology and tumour development in other tissues. For example, miR‐9 is highly expressed in primary breast cancers, where it promotes angiogenesis and metastasis (Ma *et al*., 2010). In contrast, downregulation of miR‐9 has been reported in other cancer types, such as leukaemias (Emmrich *et al*., 2014), suggesting cell‐ and tumour‐specific effects that might be explained by different expression levels of its targets.

Cellular senescence is a highly stable cell cycle arrest triggered by replicative exhaustion or in response to different stresses (Collado *et al*., 2007; Kuilman *et al*., 2010). Senescence was first described *in vitro*, but its relevance as a physiological process *in vivo* is increasingly clear. Senescence induced in response to oncogenes has been recognized as a tumour suppressor mechanism (Kuilman *et al*., 2010; Perez‐Mancera *et al*., 2014). In addition, senescence also has roles in early embryogenesis and aging amongst other physiological processes (Munoz‐Espin & Serrano, 2014). The *INK4/ARF* locus encodes three proteins involved in the implementation of senescence: the cyclin‐dependent kinase inhibitors (CDKI) p16^INK4a^ and p15^INK4b^ and ARF, a regulator of p53 (Gil & Peters, 2006; Kim & Sharpless, 2006). In proliferating cells, the expression of the *INK4/ARF* locus is tightly controlled by the action of Polycomb repressive complexes (PRCs). The PRC1 complex is comprised of four core subunits, which include orthologues of Drosophila Polycomb, Posterior sex combs, Polyhomeotic and Sex combs extra (Simon & Kingston, 2009). CBX7 is one of five mammalian orthologues of Drosophila Polycomb. As a component of PRC1, CBX7 represses the *INK4/ARF* locus (Gil & O'Loghlen, 2014). Indeed, CBX7 was first identified in a screen for bypass of replicative senescence (Gil *et al*., 2004). More recently, CBX7 was recognized as the main orthologue of *Drosophila* Polycomb implicated in maintaining the self‐renewal of embryonic stem (ES) cells (Morey *et al*., 2012; O'Loghlen *et al*., 2012). In ES cells, the miR‐125 and miR‐181 families regulate CBX7 expression levels and impact on the balance between self‐renewal and differentiation (O'Loghlen *et al*., 2012).

Little is known about how the expression and function of CBX7 are regulated. Here, we report that the miR‐9 family of miRNAs regulates CBX7 expression and controls cellular senescence. As part of a negative regulatory feedback loop, CBX7 regulates miR‐9 by binding to its promoter region and repressing its transcription. The miR‐9/CBX7 feedback loop could behave as a regulatory module contributing to upregulate p16^INK4a^ at the onset of senescence.

## Results

### miR‐9 regulates CBX7

**Figure 1:**
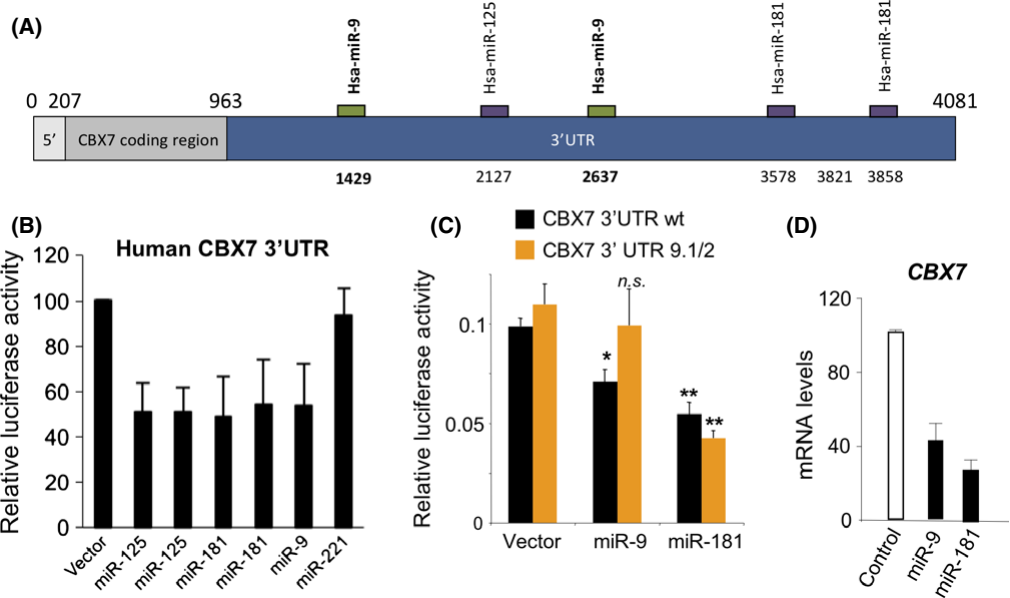
miR‐9 targets human CBX7. (A) Schematic representation of CBX7 mRNA. Bioinformatics analysis for miRNAs targeting the 3′UTR of human CBX7 was performed using manual analysis and the Targetscan and microRNA.org resources. Potential target sites for miR‐125, miR‐9 and miR‐181 in the 3′UTR of human CBX7 are indicated. Nucleotide positions refer to the ENST00000216133 transcript. (B) A luciferase reporter assay shows that miR‐9 also regulates the 3′UTR of human CBX7. (C) Mutation of two sites in the 3′UTR makes CBX7 3′UTR reporter resilient to miR‐9 regulation. A luciferase reporter assay similar to (B) was carried out using either a CBX7 3′UTR wt reporter (black bars) or a CBX7 3′UTR 9.1/2 reporter (with two miR‐9 sites mutated, orange bars). Statistical significance was calculated using two‐tailed Student's *t*‐tests, **P < 0.01; *P < 0.05; n.s. nonsignificant. (D) Transfection of a miR‐9 mimic downregulates CBX7 expression, as shown by qRT–PCR. miR‐181 is used as a positive control for downregulating CBX7.

We recently carried out a screen to identify miRNAs that regulate CBX7 using a mouse Cbx7 3′UTR reporter and a miRNA expression library comprising 371 miRNAs (O'Loghlen *et al*., 2012). The screen yielded miRNAs belonging to the miR‐125 and miR‐181 families, and their ability to modulate CBX7 was confirmed in a number of ways (O'Loghlen *et al*., 2012). We subsequently took advantage of target prediction software (targetscan and microRNA.org) (Betel *et al*., 2008; Friedman *et al*., 2009), which in addition to correctly predicting the target sites for miR‐125 and miR‐181 in CBX7 identified a potential target site for miR‐9 in the 3′UTR. Manual analysis identified a putative second target site for miR‐9 (Fig. 1A). We decided to investigate whether miR‐9 regulates CBX7 using a reporter vector in which the 3′UTR of human CBX7 is inserted into a luciferase reporter vector (psiCHECK2‐CBX7‐3′UTR). Expression of miR‐9 repressed the luciferase activity of the reporter suggesting that miR9 did indeed target human CBX7 (Fig. 1B). Having previously identified miR‐181 as a regulator of CBX7 in senescence and stem cell regulation (O'Loghlen *et al*., 2012), we used it as a control for subsequent experiments. Next, we mutated the two miR‐9 sites to generate the CBX7‐3′UTR 9.1/2 reporter. While miR‐181 was still able to downregulate the CBX7‐3′UTR 9.1/2 reporter, miR‐9 expression did not decrease luciferase activity, suggesting that miR‐9 regulates CBX7 expression by targeting these sites in the 3′UTR (Fig. 1C). We confirmed that the vectors used resulted in expression of miR‐9 and miR‐181, respectively (Fig. S1). In addition, transient transfection of IMR90 cells with either synthetic miR‐9 or miR‐181 mimetics caused a reduction in the endogenous CBX7 levels as determined by qRT–PCR (Fig. 1D). A similar result was observed after infecting IMR90 cells with retroviral vectors encoding the miR‐9 and miR‐181 precursors (data not shown). Together, these results suggest that miR‐9 regulates CBX7 expression.

### CBX7 regulates *miR‐9‐1* and *miR‐9‐2*

**Figure 2:**
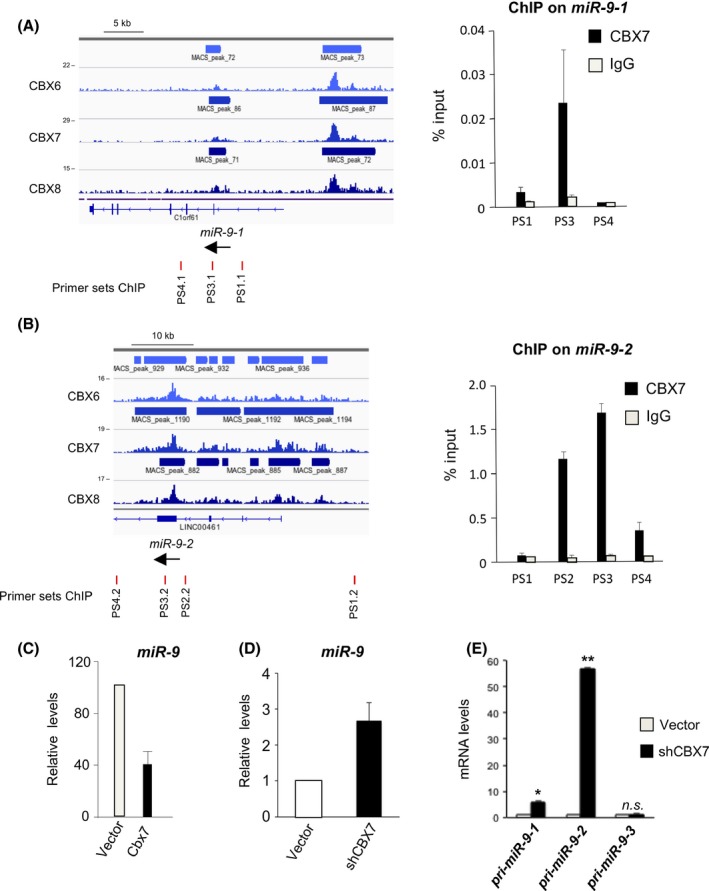
CBX7 represses the expression of *miR‐9‐1* and *miR‐9‐2*. (A, B) CBX6, CBX7 and CBX8 associate with miR‐9‐1 (A) and miR‐9‐2 (B). ChIP‐seq for CBX6, CBX7 and CBX8 was performed in Hs68 human fibroblasts (left panel). Their binding profiles on miR‐9‐1 (A) and miR‐9‐2 (B) are shown. The location of primer sets used (primer sets PS1.1, PS3.1 and PS4.1 for miR‐9‐1 and PS1.2, PS2.2, PS3.2 and PS4.2 for miR‐9‐2) is also depicted (left). ChIP in human fibroblast cells confirming that CBX7 binds to miR‐9‐1 (A) and miR‐9‐2 (B) is shown (right). (C) CBX7 overexpression reduces miR‐9 levels in IMR90 cells as shown by qRT–PCR. (D) CBX7 knockdown results in an increase in miR‐9 as assessed by qRT–PCR. (E) Knockdown of CBX7 results in upregulated levels of pri‐miR‐9‐1 and pri‐miR‐9‐2 as assessed by qRT–PCR. Statistical significance was calculated using two‐tailed Student's *t*‐tests, **P < 0.01; *P < 0.05; n.s. nonsignificant.

MicroRNAs are involved in fine‐tuning patterns of gene expression, often through regulatory feedback mechanisms between the miRNAs and their target genes (Zhao *et al*., 2009; Overhoff *et al*., 2014). To test whether CBX7 also regulates miR‐9, we analysed chromatin immunoprecipitation (ChIP)‐Seq data sets that we have reported elsewhere (Pemberton *et al*., 2014). ChIP‐Seq of various PRC1 components, including CBX7, revealed binding at two of the three loci coding for miR‐9 family genes (Fig. 2A,B). The three microRNAs, miR‐9‐1, 9‐2 and 9‐3, are encoded by distinct loci on chromosomes 1, 5 and 15, respectively, and share the same mature sequence. Little is known about miR‐9‐3, and it did not register as a PRC1 target in the ChIP‐Seq analysis. Additional ChIP experiments using qPCR and primer sets that span the relevant genomic DNA confirmed that CBX7 binds specifically to the regions adjacent to or upstream of miR‐9‐1 and miR‐9‐2 (Fig. 2A,B). These results implied that CBX7 could be transcriptionally repressing miR‐9 expression. Consistent with this idea, over‐expression of mouse Cbx7 caused down‐regulation of miR‐9 (Fig. 2C, Fig. S2A), whereas shRNA‐mediated knockdown of CBX7 resulted in up‐regulation of miR‐9 (Fig. 2D, Fig. S2B). To further confirm these results, we looked into how knockdown of CBX7 affected the expression of the primary miRNA transcripts (pri‐miRNAs) of the miR‐9 family. Confirming the ChIP data, knockdown of CBX7 resulted in upregulation of pri‐miR‐9‐1 and pri‐miR‐9‐2, without affecting pri‐miR‐9‐3 expression (Fig. 2E). These results imply the existence of an autoregulatory network between miR‐9 and CBX7, where miR‐9 downregulates CBX7 and CBX7 transcriptionally represses miR‐9.

### The expression of miR‐9 is induced during replicative senescence

**Figure 3:**
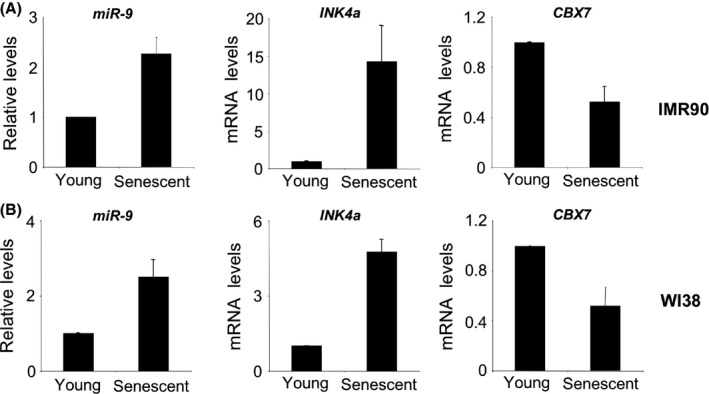
miR‐9 expression is upregulated during replicative senescence of human fibroblast. The levels of *miR‐9* (left), *INK4a* mRNA (coding for p16^INK^ ^4a^, centre) and *CBX7* mRNAs (right) were analysed by qRT–PCR comparing either IMR90 (A) or WI38 (B) young and senescent cells.

Next, we investigated the expression of miR‐9 during senescence. To this end, we used two different human fibroblast strains, IMR90 and WI38. Using RNA obtained from low passage (young) and late passage (senescent) fibroblasts, we confirmed that the expression of the mRNA encoding for p16^INK4a^ increased during senescence, as expected. This correlated with a decrease in CBX7 mRNA levels and an induction of miR‐9 in both fibroblast strains (Fig. 3). The above results suggest that miR‐9 might have a role in fine‐tuning CBX7 expression during replicative senescence.

### The expression of miR‐9 causes senescence

**Figure 4:**
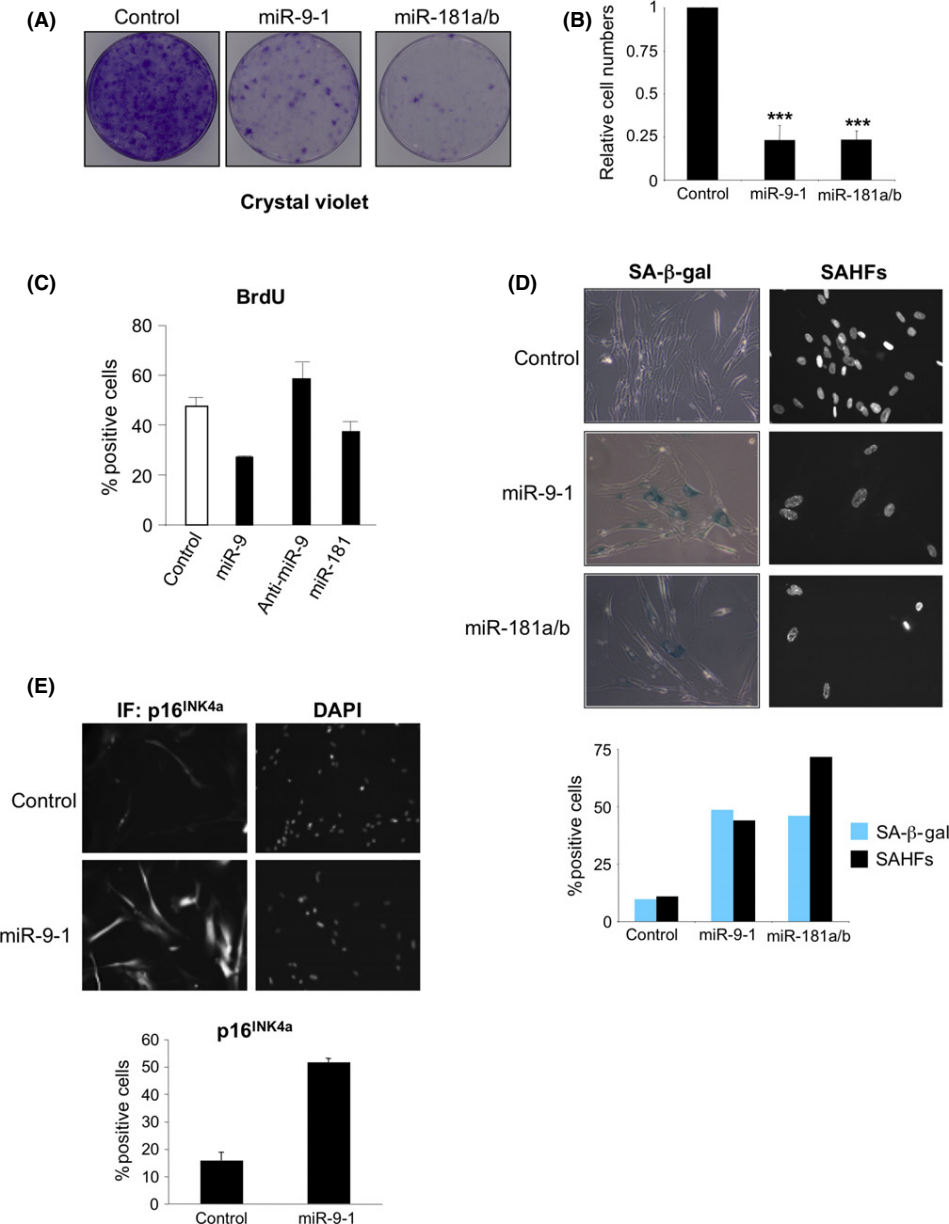
miR‐9 expression triggers cellular senescence. (A, B) Overexpression of miR‐9‐1 results in decreased proliferation of IMR90 cells. IMR90 cells infected with miR‐9‐1 (or miR‐181a/b, or control vectors) were selected, and cells seeded at low density. Plates were stained with crystal violet 2 weeks later. (A) Representative plates and (B) quantification of crystal violet staining (average of five independent experiments) are shown. Statistical significance was calculated using two‐tailed Student's *t*‐tests, ***P < 0.001. (C) miR‐9 triggers arrest of IMR90 cells. BrdU incorporation was assessed by IF in IMR90 cells transfected with miR‐9 or miR‐181 mimics or with anti‐miR‐9. (D) IMR90 cells infected with the indicated vectors were subjected to SA‐β‐Gal (left) and DAPI staining (right) to visualize SAHFs. Representative images (top) and quantification of the percentages positive for SA‐β‐Gal or containing SAHFs are shown (bottom). (E) Expression of miR‐9 induces p16^INK^ ^4a^. The levels of p16^INK^ ^4a^ were assessed by quantitative IF in IMR90 cells infected with a retroviral vector expressing miR‐9.

Given that miR‐9 expression resulted in the downregulation of CBX7 mRNA levels, and CBX7 has been linked with senescence regulation, we decided to test the effect that miR‐9 has on senescence. Infection of IMR90 cells with a retroviral vector encoding miR‐9‐1 led to reduced cell growth, analogous to the effects of miR‐181. This manifested as a decrease in colony formation as judged by crystal violet staining (Fig. 4A) and reduced cell numbers (Fig. 4B). Similarly, transfection of synthetic miR‐9 oligomers caused a decrease in cell numbers (data not shown) and BrdU incorporation, whereas antagomirs targeting miR‐9 had the converse effect (Fig. 4C). To assess whether this arrest has characteristics of senescence, we infected IMR90 cells with a vector encoding miR‐9‐1 and observed an increase in the percentage of cells that were positive for SA‐β‐Gal staining and also an increase in the percentage of cells presenting senescence‐associated heterochromatin foci (SAHFs, Fig. 4D), but did not detect a significant increase in the induction of SASP components (Fig. S3A). However, we did not detect significant cell death (data not shown) suggesting senescence. To explore how miR‐9 caused senescence, we determined the effect that miR‐9 had on the expression of key senescence effectors such as p16^INK4a^, p21^CIP^ and p53 using immunofluorescence and high‐throughput microscopy. Expression of miR‐9 in IMR90 cells induced p16^INK4a^ (Fig. 4E) without affecting p21^CIP^ (Fig. S3B) or p53 (Fig. S3C). The induction of p16^INK4a^ by miR‐9 was confirmed by transfecting synthetic miR‐9 (Fig. S3D). Together, these results suggest that increased miRNA‐9 levels alter the equilibrium of the miR9/CBX7 regulatory loop resulting in upregulation of p16^INK4a^ and senescence.

### miR‐9 induces senescence in a CBX7‐dependent fashion

**Figure 5:**
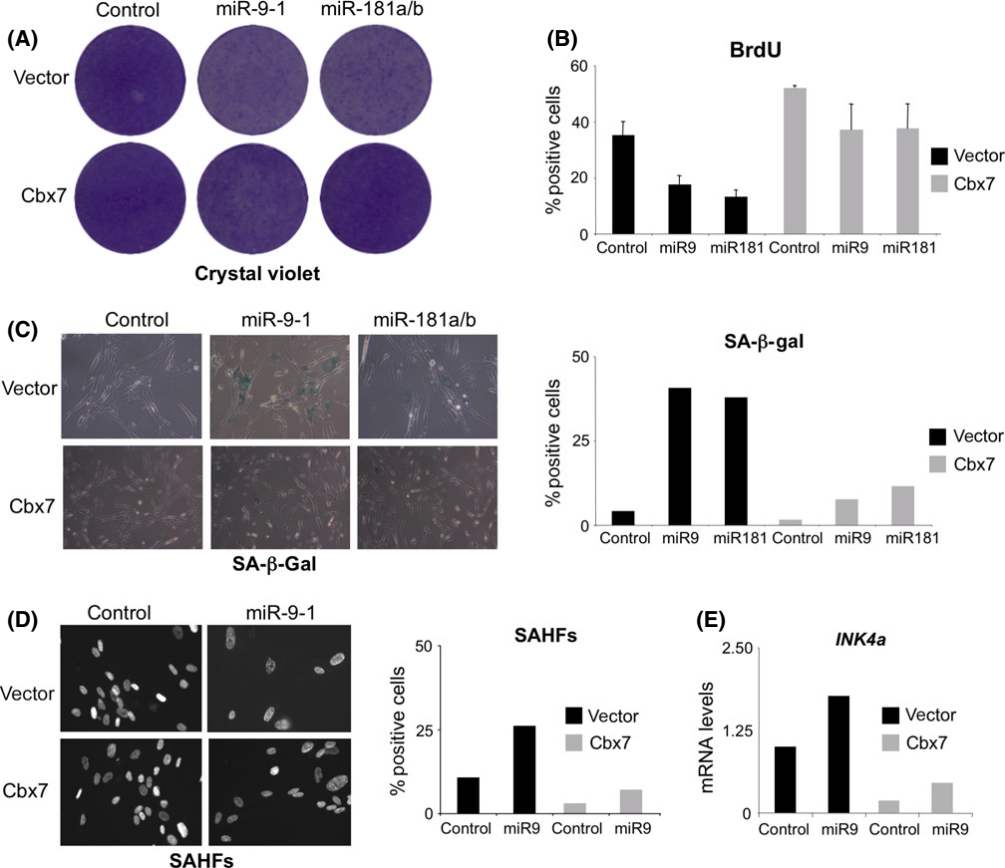
miR‐9‐induced senescence is rescued by CBX7 overexpression. (A) Control and IMR90 cells overexpressing murine CBX7 (Cbx7) were infected with pMSCV‐miR‐9‐1, miR‐181a/b or control vectors, cells selected and seeded at low density. Crystal violet stained plates are shown. (B–D) Cells derived from the same experiments were used to measure (B) BrdU incorporation, (C) SA‐β‐Gal positive cells and (D) the percentage of cells showing SAHFs. (E) The levels of the *INK4a* transcript (encoding for p16^INK^ ^4a^) were analysed by qRT–PCR in the indicated cells.

To substantiate the role of CBX7 and p16^INK4a^ in miR‐9‐induced senescence, we first generated IMR90 cells overexpressing murine CBX7 (Cbx7). Expression of miR‐9 or miR‐181 resulted in a significant arrest (Fig. 5A) and a decrease in BrdU incorporation (Fig. 5B) in the control cells, but not in cells overexpressing CBX7. Notably, while expression of miR‐9 increased the percentage of IMR90 cells positive for SA‐β‐Gal (Fig. 5C) and SAHF (Fig. 5D), expression of CBX7 blunted the ability of miR‐9 to induce senescence. Importantly, these phenotypes correlated with decreased *INK4a* levels in cells overexpressing CBX7 (Fig. 5E).

### The arrest caused by miR‐9 is dependent on p16^INK4a^

**Figure 6:**
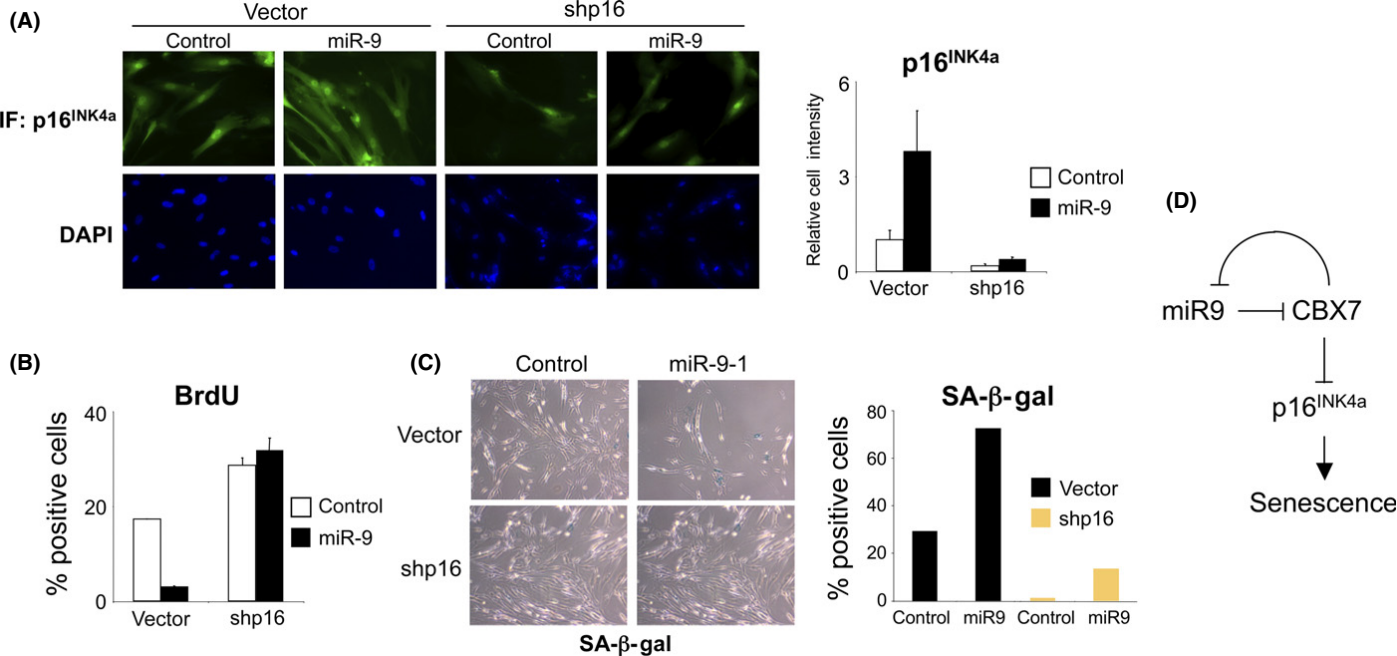
miR‐9‐induced senescence is p16^INK^ ^4a^‐dependent. (A) IMR90 cells were infected with a vector knocking down p16^INK^ ^4a^ expression (shp16) or the corresponding control (vector). The derived cells were subsequently infected with viruses expressing miR‐9 or controls. The expression of p16^INK^ ^4a^ was assessed by IF. Representative pictures (left) and quantification (right) is shown. (B, C) Knocking down p16^INK^ ^4a^ expression suppressed miR‐9‐induced senescence. The percentage of (B) cells incorporating BrdU and (C) cells positive for SA‐β‐Gal staining is shown. (D) Scheme depicting the regulatory network formed by CBX7 and miR‐9 to control p16^INK^ ^4a^ expression and regulate senescence.

Next, we took advantage of the Leiden strain of human fibroblasts that carry a mutation that functionally inactivates both copies of p16^INK4a^ (Brookes *et al*., 2002). Immunostaining revealed that the levels of the altered protein in Leiden fibroblasts are lower than those of wild‐type p16^INK4a^ in IMR90 cells (Fig. S4A). The effect that miR‐9 expression had on proliferation of IMR90 fibroblasts, as evaluated by crystal violet staining (Fig. S4B) and BrdU incorporation (Fig. S4B), was mitigated in the Leiden strain of fibroblasts, suggesting that they depended on p16^INK4a^. To confirm that p16^INK4a^ mediates the arrest caused by miR‐9, we derived cells in which p16^INK4a^ expression was knocked down taking advantage of shRNA (Fig. 6A). While expression of miR‐9 in IMR90 cells caused growth arrest (Fig. 6B) and increased the number of SA‐β‐Gal positive cells (Fig. 6C), the same was not observed in IMR90 shp16 cells. Taken together, these findings imply that miR‐9 and CBX7 are involved in a regulatory network that controls p16^INK4a^ expression to regulate senescence.

## Discussion

In recent years, there has been considerable progress in understanding the mechanisms by which PRCs are recruited to their target genes and regulate gene expression (Luis *et al*., 2012; Gil & O'Loghlen, 2014). Much less is known about how the function and expression of the PRCs and their components are controlled. The regulation of CBX7 is particularly interesting because CBX7 is implicated in senescence (Gil *et al*., 2004) and cancer (Bernard *et al*., 2005; Scott *et al*., 2007; Pallante *et al*., 2008; Karamitopoulou *et al*., 2010). In addition, CBX7 levels and consequently the composition of PRC1 complexes change dramatically during ES cell differentiation (Morey *et al*., 2012; O'Loghlen *et al*., 2012). In a screen for transcription factors that regulate CBX7 (O'Loghlen *et al*., 2015), we identified E2F family members and the nuclear receptor NR2E1 as positive regulators of CBX7 expression (O'Loghlen *et al*., 2015). Previously, we also described how miRNAs of the miR‐125 and miR‐181 families negatively regulate CBX7 during ESC differentiation (O'Loghlen *et al*., 2012). Here, we add miR‐9 as another miRNA family that contributes to the regulation of CBX7. Although our results suggest that miR‐9 directly controls the stability of CBX7 mRNA, further experiments are needed to fully discard an indirect regulation or miR‐9 inhibiting the translation of CBX7.

Interestingly, miR‐9 levels are upregulated during replicative senescence, suggesting it might contribute to fine‐tune the induction of p16^INK4a^ in that context. Ectopic expression of miR‐9 induces a senescence‐like arrest, dependent on p16^INK4a^ induction (Fig. 6D). Cells expressing miR‐9 became arrested and SA‐β‐Gal positive, developed SAHFs and upregulated p16^INK4a^ expression. However, we could not detect an activation of p53 or p21 or significant induction of several SASP components. This is reminiscent of the arrest caused by JMJD3 overexpression, which also results in p16^INK4a^ induction in the absence of activation of p53 (Barradas *et al*., 2009). Moreover, it has been described that p16^INK4a^ induction is sufficient to trigger senescence in the absence of SASP (Coppe *et al*., 2011), similar to what we observe upon miR‐9 expression.

MicroRNAs often fine‐tune patterns of gene expression as part of regulatory feedback loops; the aforementioned regulation of CBX7 by miR‐181 and miR‐125 in ESCs is such an example (O'Loghlen *et al*., 2012). Similarly, a study on human mammary epithelial cells (HMECS) identified several miRNAs that regulate p16^INK4a^ expression (Overhoff *et al*., 2014) and suggested that they might target different components of PRC1 and PRC2. In addition, it has been shown that the miRNA let‐7 can downregulate HMGA2 levels and is responsible for the induction of p16^INK4a^ during the aging of adult stem cells (Nishino *et al*., 2008). Here, we described an autoregulatory loop involving CBX7 and miR‐9 that could contribute to fine‐tune the induction of p16^INK4a^ during replicative senescence.

The miR‐9 family affects different physiological processes. miR‐9 can inhibit cell proliferation and migration in the context of cancer (Selcuklu *et al*., 2012; Yu *et al*., 2013), although its effects can be both cell type and context dependent (Ma *et al*., 2010). miR‐9 may also have a role during aging. While miR‐9 can target prelamin A, thereby preventing the expression of progerin in models of Hutchinson‐Gilford progeria syndrome (HPGS) (Jung *et al*., 2012), other studies (Olivieri *et al*., 2013) and this report have observed that the expression of miR‐9 can increase during senescence. The ability of miR‐9 to downregulate CBX7 expression causing p16^INK4a^ induction and senescence could therefore provide an explanation for the role of miR‐9 in cancer and aging.

As with many other miRNAs, miR‐9 targets multiple genes. Some of these targets can potentially explain the physiological effects of miR‐9. For example, the ability of miR‐9 to inhibit cell proliferation has been linked to downregulation of MTHFD2 expression in cancer cells (Selcuklu *et al*., 2012) or TLX/NR2E1 in neural stem cells (Liu *et al*., 2010; Zhu *et al*., 2014). The regulation of CXCR4 by miR‐9 has also been invoked to explain the effect of miR‐9 on cell growth (Yu *et al*., 2013). In addition, miR‐9 can regulate *NF‐KB1*, affecting cell migration (Liu *et al*., 2012), and E–cadherin (Ma *et al*., 2010), explaining its role in regulating metastatic growth. It would clearly be interesting to determine how the regulatory module involving CBX7 and miR‐9 contributes to these physiological processes and whether it influences aging.

## Experimental procedures

### Cell culture and retroviral infection

HEK293T, Hs68, WI38 and IMR90 cells were obtained from the ATCC. Leiden human fibroblasts have been previously described (Brookes *et al*., 2002). Cells were maintained in Dulbecco's modified Eagle's medium (Invitrogen, Paisley, UK) with 10% foetal bovine serum (PAA, Amersham, UK) and 1% antibiotic–antimycotic solution (Invitrogen, Paisley, UK). Methods used for retrovirus production and infection have been described previously (Acosta *et al*., 2008).

### Plasmids

Plasmids encoding for different microRNAs and its corresponding control were obtained from the miR‐Vec library (Voorhoeve *et al*., 2006). The CBX7 reporter plasmid has been described before (O'Loghlen *et al*., 2012) and was used to derive the CBX7 3′UTR 9.1/2 reporter by Gibson assembly. Retroviral vectors for CBX7 overexpression and for shRNA‐mediated knockdown of CBX7 and p16 have been described previously (O'Loghlen *et al*., 2012).

### miRNA reverse transfection and luciferase assay

For the luciferase assay, HEK293T cells were reverse transfected using polyethylenimine (PEI; Sigma, St Louis, MO, USA) to individually transfect our positive clones from the miR library in a 96‐well plate format. A 9:1 ratio of miR‐Vec to luciferase reporter construct was used. miR‐Vec‐Ctrl was used as control vector. A 3:1 ratio of PEI to DNA was used, and after incubation of reagent–DNA complexes for 30 min, cells were added. Firefly and Renilla luciferase activities were measured using the Dual‐Luciferase Reporter Assay system (Promega, Madison, WI, USA) 48 h after transfection.

### miRNA transfection

IMR90 cells were transfected with 30 nm miRNA or anti‐miRNA antagomir in 6‐well plates. A 3.5% solution of HiPerFect transfection reagent (QIAGEN, Valencia, CA, USA) was prepared in serum‐free DMEM and then mixed with the miRNA or anti‐miRNA. The mix was incubated for 30 min at room temperature and then added to the cells. Medium was changed on the following day, and cells were either fixed for immunofluorescence or harvested for RNA extraction 24–96 h later. A scrambled siRNA (AllStars) or Silencer Select Negative Control #1 and #2 siRNA (Ambion, Carlsbad, CA, USA) were included as negative controls in most experiments.

### Quantitative RT–PCR analysis

Total RNA and miRNAs were extracted using miRCURY RNA isolation kit (Exiqon, Vedbaek, Denmark) or High Pure miRNA Isolation kit and High Pure RNA Isolation Kit (Roche, Basel, Switzerland). cDNAs were generated using SuperScript II reverse transcriptase (Invitrogen, Paisley, UK). For miRNA reverse transcription, TaqMan miRNA reverse transcription kit was used with specific miRNA primers. PCR reactions were performed in an Opticon 2 Real‐Time PCR Detection System (Biorad, Hercules, CA, USA) using Power SYBR Green Master Mix or TaqMan Universal PCR Master Mix (Applied Biosystems, Carlsbad, CA, USA). Expression was normalized to human ribosomal protein S14 (RPS14) or Glyceraldehyde 3‐phosphate dehydrogenase (GAPDH) for mRNAs and RNU6B or U6snRNA for miRNAs. Taqman probes used in this study were acquired from Life Technologies and include CBX7 (Hs00545603_m1), TBP (4333769F), Hsa‐miR‐9 (A583), RNU6B (1093), U6snRNA (1973). Primers used for RT–PCR are as follows: mouse Cbx7 (For: GGATGGCCCCCAAAGTACAG; Rev: TATACCCCGATGCTCGGTCTC), INK4a (For: CGGTCGGAGGCCGATCCAG; Rev: GCGCCGTGGAGCAGCAGCAGCT), IL‐8 (For: GAGTGGACCACACTGCGCCA; Rev: TCCACAACCCTCTGCACCCAGT), IL‐6 (For: CCAGGAGCCCAGCTATGAAC; Rev: CCCAGGGAGAAGGCAACTG), CXCL1 (For: GAAAGCTTGCCTCAATCCTG; Rev: CACCAGTGAGCTTCCTCCTC), RPS14 (For: TCACCGCCCTACACATCAAACT; Rev: CTGCGAGTGCTGTCAGAGG), Pri‐miR9‐1 (For ACTGTGACTCCTACCTGTGC; Rev ATAACCCCATACACTGCGCA), Pri‐miR9‐2 (For: TGCCGGAGATTACTTGCTGA; Rev: TTCCTCTTGCCAGACTCCAG), Pri‐miR9‐3 (For: CCACAGAGCCGTCATAAAGC; Rev CAGGAAAGAGGAGGACTGGG), GAPDH (For: ACCACAGTCCATGCCATCAC; Rev: TCCACCACCCTGTTGCTGTA).

### Chromatin immunoprecipitation

Chromatin immunoprecipitation experiments were performed as described previously (Pemberton *et al*., 2014). Immunoprecipitation of cross‐linked chromatin was conducted with CBX7 antibody (ab21873; Abcam, Cambridge, UK). After immunoprecipitation, DNA was extracted using the QIAquick PCR purification kit (Qiagen) and an aliquot amplified by real‐time qPCR using the following primers: hsa‐miR‐9‐1. PS1.1 (For: TTCTCGAATGCTGTGGACTG; Rev: AGAAGACGGTCTGGAAAGCA); PS3.1 (For: GCGCAGTGTATGGGGTTATT; Rev: GCGGGGTTGGTTGTTATCTT); PS4.1 (For: TGTCTGTGTGCCTGAAGAGG; Rev: GAATCCACCCTTTCCCAAAT). Hsa‐miR‐9‐2. PS1.2 (For: CTGCCAAATCATCAGCTTCA; Rev: TTCCTCCCATTTCAGTCTGG); PS2.2 (For: AGGCCGCTTTACAGGGTTAT; Rev: GCAAATACATTGCCCGAGTT); PS3.2 (For: GCCTCCCCTCTTGTCAAAGT; Rev: AGGCAAGACAGACCCTCAGA); PS4.2 (For: ATGACAGGGCCAATGAG; Rev: CCGAGGGCCAGTGACTATTA). To confirm target enrichment, each PCR product was evaluated first by standard end point PCR.

### ChIP‐seq and bioinformatics analysis

Parallel ChIP experiments were performed using approximately 5 μg of antibody with 500 μg chromatin. The recovered material was pooled and concentrated to a minimum of 0.2 μg μL^−1^. Input DNA was used as control for the ChIP‐seq analysis. Library preparation and Solexa genome‐wide sequencing was performed as recommended by the manufacturer. The alignments were performed using novoalign (version 2.07.14; http://novocraft.com) allowing for a single mismatch per read. Duplicates were removed using the picard markduplicates program (Picard‐tools package version 1.48; http://picard.sourceforge.net), and peak calling was performed with macs (version 1.4.0rc2; 46).

### BrdU assay and crystal violet staining

BrdU labelling was performed for 24 h. Crystal violet staining was performed as previously described (Acosta *et al*., 2008).

### SA‐β‐Galactosidase staining and SAHF quantification

SA‐β‐Galactosidase staining and quantification was performed as previously described (Acosta *et al*., 2008). For SAHF quantification, cells were stained with DAPI and the nuclei of at least 100 cells per condition were assessed.

### Immunofluorescence and immunoblotting

The following antibodies were used: BrdU (A21303; Invitrogen, Paisley, UK), p16^INK4a^ (JC‐8, CRUK), p53 (sc‐126, DO1; Santa Cruz Biotechnology, Wembley, UK) and p21^CIP^ (CP74, Sigma). Immunofluorescence was performed using an automated high‐throughput microscope (InCell Analyzer 1000; GE Healthcare, Amersham, UK). Image processing and quantification was performed using incell investigator software (GE).

### miRNA target prediction

Bioinformatics miR prediction on the 3′UTR of human CBX7 was performed using the TargetScan (http://www.targetscan.org) and MicroRNA.org (http://www.microrna.org/microrna/home.do) websites.

## Author contributions

AO, NM, VR and SB performed the experiments and analysed the data. AO, GP and JG designed the experiments and wrote the manuscript.

## Funding

No funding information provided.

## Conflict of interest

None declared.

## Supporting information


**Fig. S1** Retroviral vectors express miR‐9 and miR‐181.
**Fig. S2** Controls for CBX7 overexpression and knockdown.
**Fig. S3** Characterization of the miR‐9 induced arrest.
**Fig. S4** Analysis of p16^INK4a^‐deficient Leiden fibroblasts suggests that miR‐9‐induced senescence is p16^INK4a^‐dependent.
