# Erratum

**DOI:** 10.1111/acel.12554

**Published:** 2017-03-03

O'Loghlen A, Brookes S, Martin N, Rapisarda V, Peters G and Gil J (2015) CBX7 and miR‐9 are part of an autoregulatory loop controlling p16INK4a. Aging Cell, 14: 1113–1121. doi: 10.1111/acel.12404

In the article ‘CBX7 and miR‐9 are part of an autoregulatory loop controlling p16^INK4a^’, the two lower panels of Figure 6C are duplicated. The correct Figure 6 with the new shp16/control image in panel C is shown below.

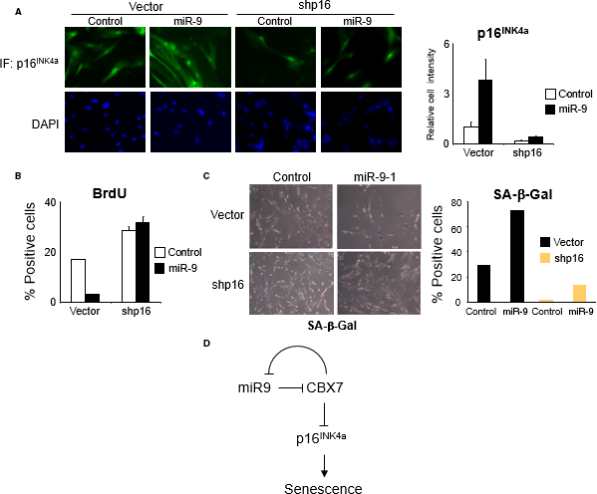

The authors would like to apologize for any inconvenience caused.

