# Supplementary material for: CBX7 and miR‐9 are part of an autoregulatory loop controlling p16INK 4a
Source: Aging Cell. 2015 Sep 29;14(6):1113–21. doi: 10.1111/acel.12404 (PMC4693451; doi:10.1111/acel.12404)
Supplement: Supplementary file 1 — Fig. S1 Retroviral vectors express miR‐9 and miR‐181. Fig. S2 Controls for CBX7 overexpression and knockdown. Fig. S3 Characterization of the miR‐9 induced arrest. Fig. S4 Analysis of p16INK4a‐deficient Leiden fibroblasts suggests that miR‐9‐induced senescence is p16INK4a‐dependent. [file ACEL-14-1113-s001.pdf]

SUPPLEMENTARY FIGURE LEGENDS

Supplementary Figure 1. Retroviral vectors express miR-9 and miR-181.

IMR90 cells were infected with the indicated vectors and the expression of miR-9 or miR-181 assessed by qRT-PCR.

Supplementary Figure 2. Controls for CBX7 overexpression and knockdown.

(A) Overexpression of mouse Cbx7 results in p16^{INK4a} repression. qRT-PCR was carried out in cells used in the experiment described in Fig 2C, to assess the levels of *INK4a* mRNA (encoding for p16^{INK4a} and the exogenously expressed mouse Cbx7 (*Cbx7*). (B) Knockdown of CBX7 results in p16^{INK4a} induction. qRT-PCR was carried out in cells used in the experiment described in Fig 2D-E, to assess *INK4a* and CBX7 mRNA levels.

Supplementary Figure 3. Characterization of the miR-9 induced arrest. (A) IMR90 cells were infected with control or miR-9 expressing vectors and the expression of the indicated genes was assessed by qRT-PCR. (B-C) The expression of (B) p21^{CIP} and (C) p53 was assessed by IF, and the percentage of positive cells were quantified. (D) miR-9 induces p16^{INK4A} expression in IMR90 cells. p16^{INK4a} expression was assessed by IF in IMR90 cells transfected with miR-9 or miR-181 mimics or with anti-miR-9.

Supplementary Figure 4. Analysis of p16^{INK4a}-deficient Leiden fibroblasts suggests that miR-9-induced senescence is p16^{INK4a}-dependent.

IMR90 and p16^{INK4a}-defective Leiden fibroblasts (Brookes *et al.* 2002) were infected with pMSCV-miR-9 or empty vector. (A) Immunofluorescence for p16^{INK4a} was performed in both cells types. Quantification (left) and representative images (right) are shown. (B) Cells were seeded at

low density and stained with crystal violet after 2 weeks. Quantification (left) and representative images (right) are shown. (C) BrdU incorporation was assessed in the indicated cells.

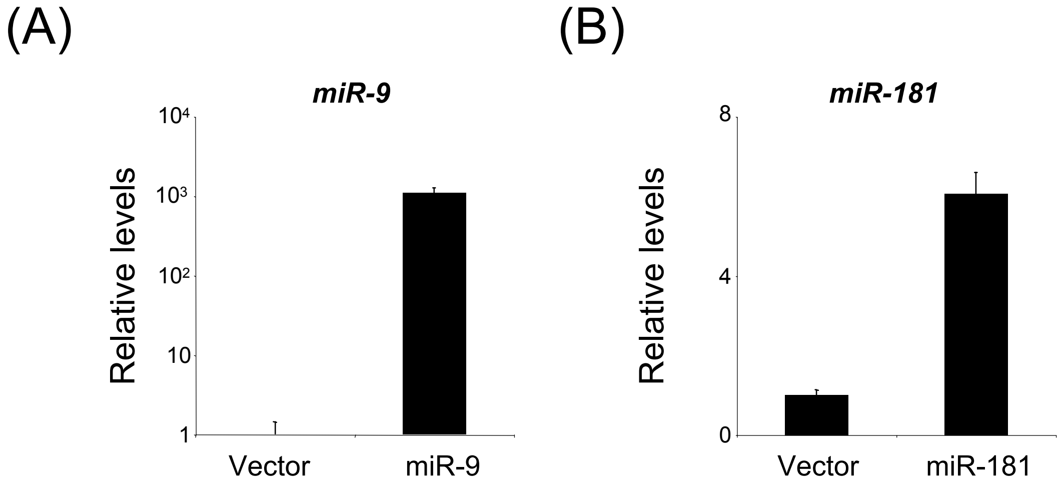
(A)
miR-9
Relative levels
10⁴
10³
10²
10
1
Vector
miR-9
(B)
miR-181
Relative levels
8
4
0
Vector
miR-181

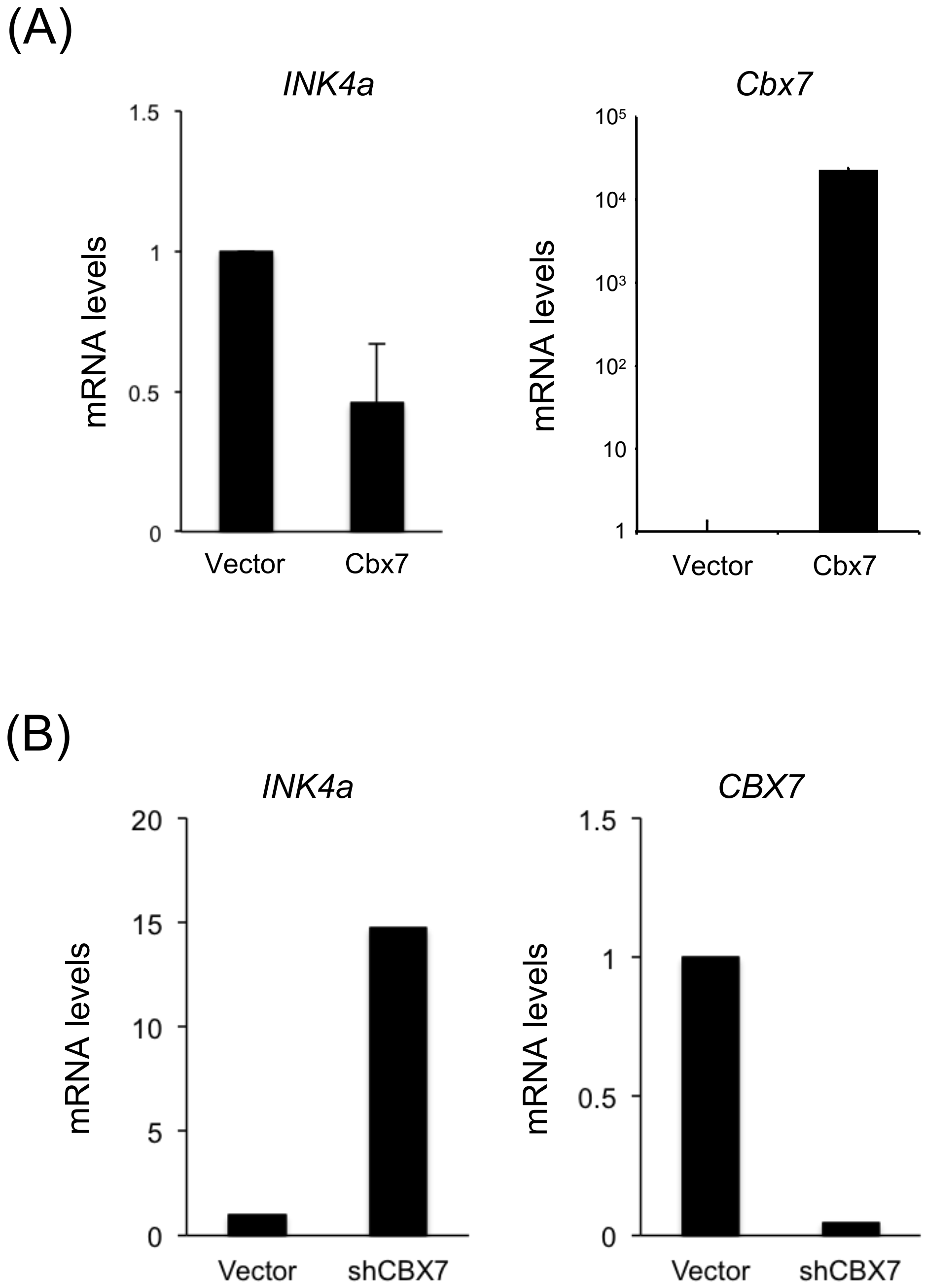
(A)
INK4a
1.5
1
0.5
0
mRNA levels
Vector
Cbx7
Cbx7
10^5
10^4
10^3
10^2
10
1
mRNA levels
Vector
Cbx7
(B)
INK4a
20
15
10
5
0
mRNA levels
Vector
shCBX7
CBX7
1.5
1
0.5
0
mRNA levels
Vector
shCBX7

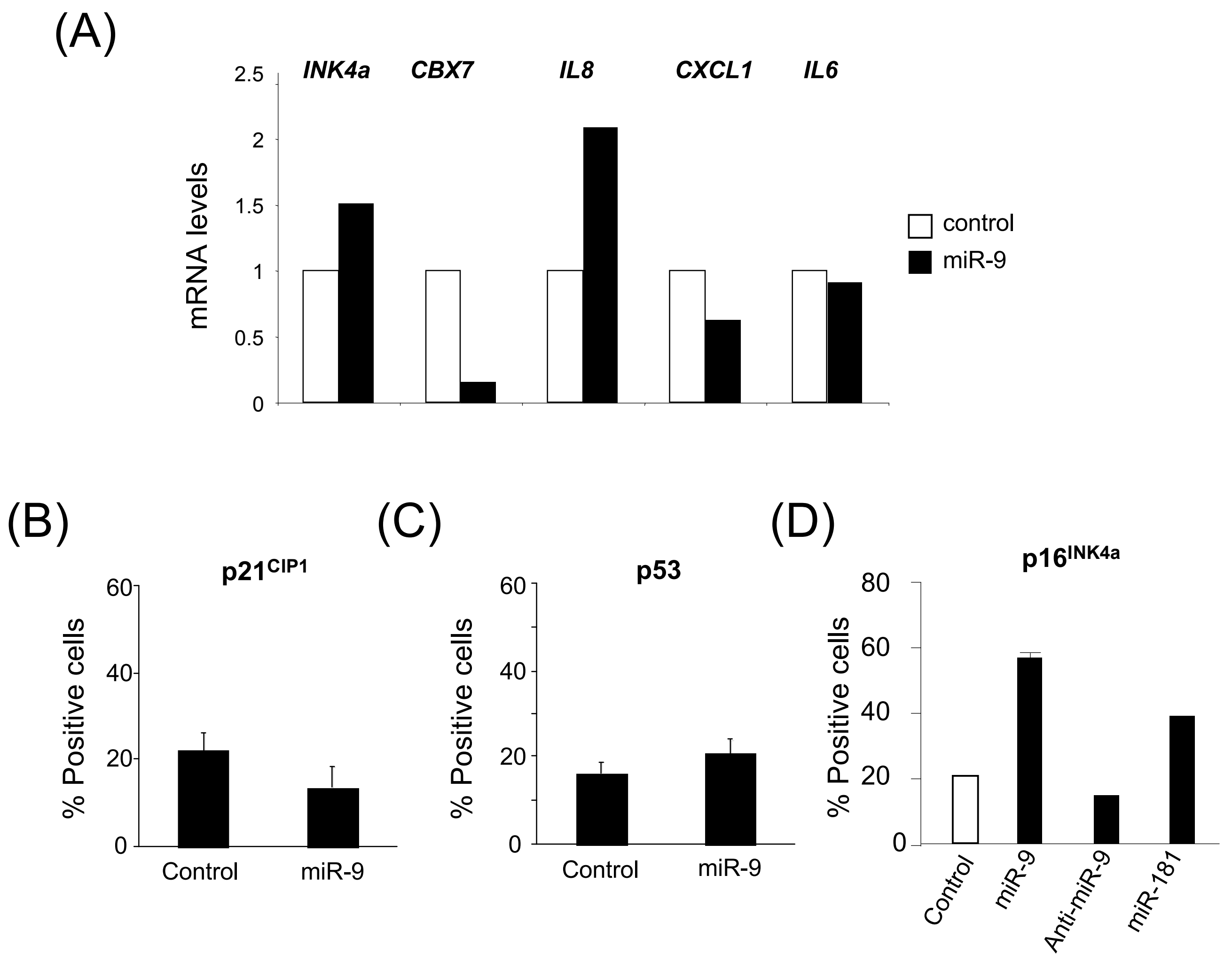

(A)
INK4a
CBX7
IL8
CXCL1
IL6
mRNA levels
2.5
2
1.5
1
0.5
0
control
miR-9
(B)
p21CIP1
% Positive cells
60
40
20
0
Control
miR-9
(C)
p53
% Positive cells
60
40
20
0
Control
miR-9
(D)
p16INK4a
% Positive cells
80
60
40
20
0
Control
miR-9
Anti-miR-9
miR-181

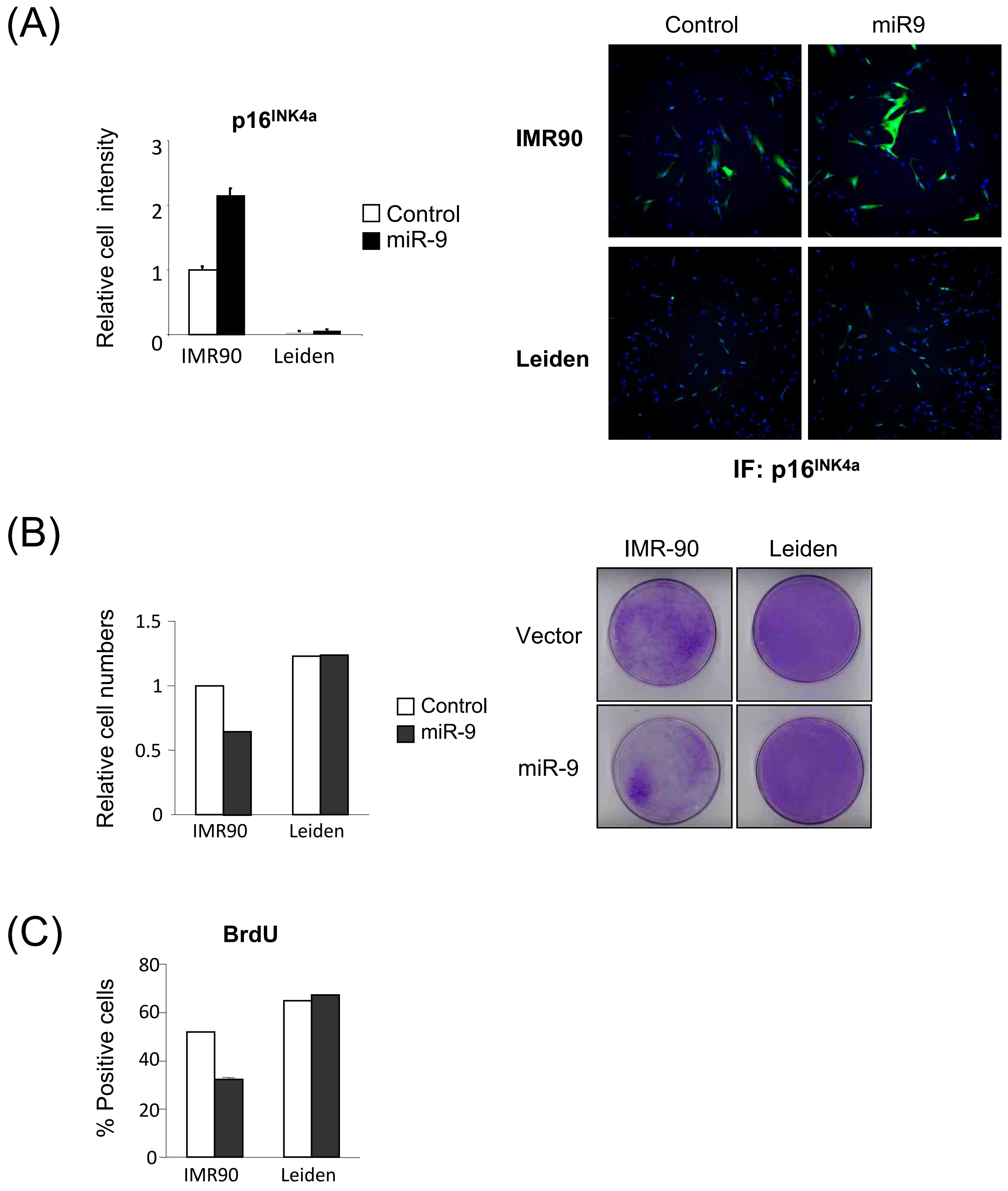

(A)
p16INK4a
Relative cell intensity
3
2
1
0
IMR90
Leiden
Control
miR-9
Control
miR9
IMR90
Leiden
IF: p16INK4a
(B)
Relative cell numbers
1.5
1
0.5
0
IMR90
Leiden
Control
miR-9
IMR-90
Leiden
Vector
miR-9
(C)
BrdU
% Positive cells
80
60
40
20
0
IMR90
Leiden
